# Supplementary material for: Genome-Wide Identification and Expression Patterns of AcSWEET Family in Pineapple and AcSWEET11 Mediated Sugar Accumulation
Source: Int J Mol Sci. 2022 Nov 10;23(22):13875. doi: 10.3390/ijms232213875 (PMC9697096; doi:10.3390/ijms232213875)
Supplement: Supplementary file 1 [file ijms-23-13875-s001.zip › supplementary figures.pdf]

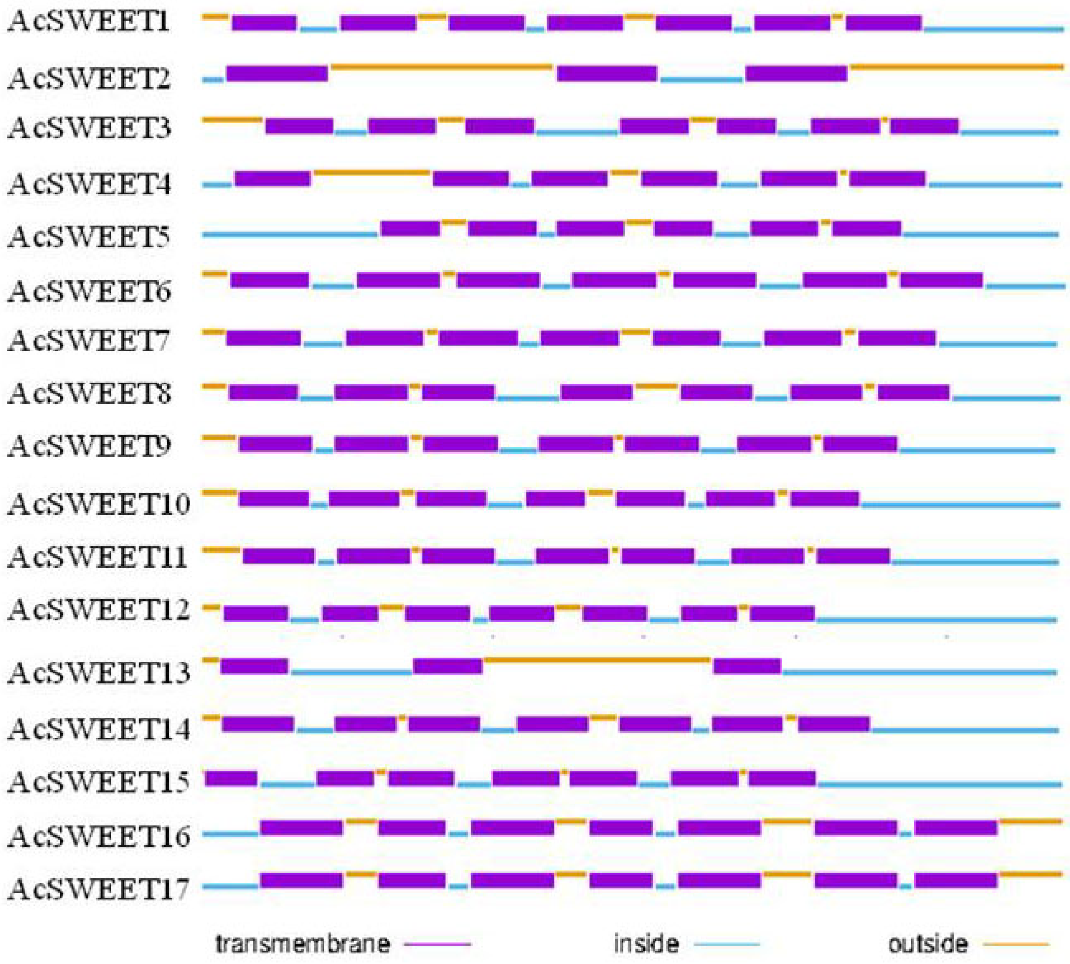

Figure S1. Protein structure of AcSWEETs in pineapple. The blue lines means the intracellular region, the purple lines indicate transmembrane, the yellow lines denotes extracellular.

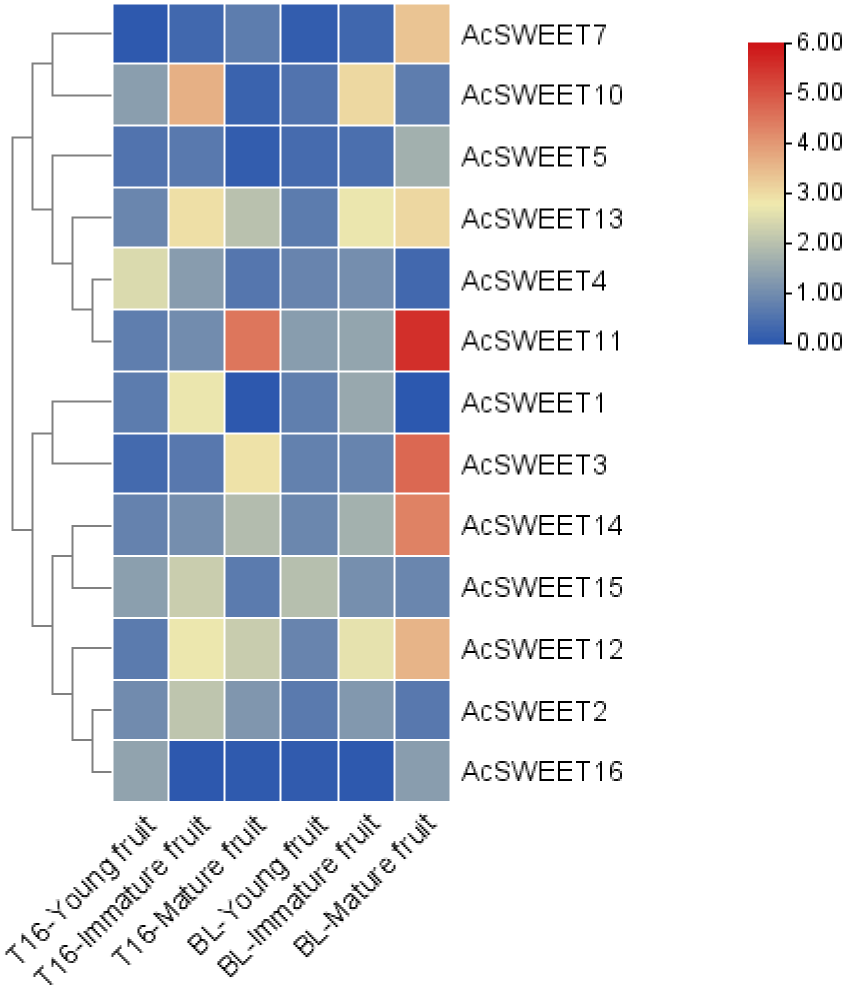

Figure S2. Expression analysis of *AcSWEETs* in pineapple fruit development of 'Tainong16'and 'Comte de paris'. T16, 'Tainong16', BL', 'Comte de paris'.

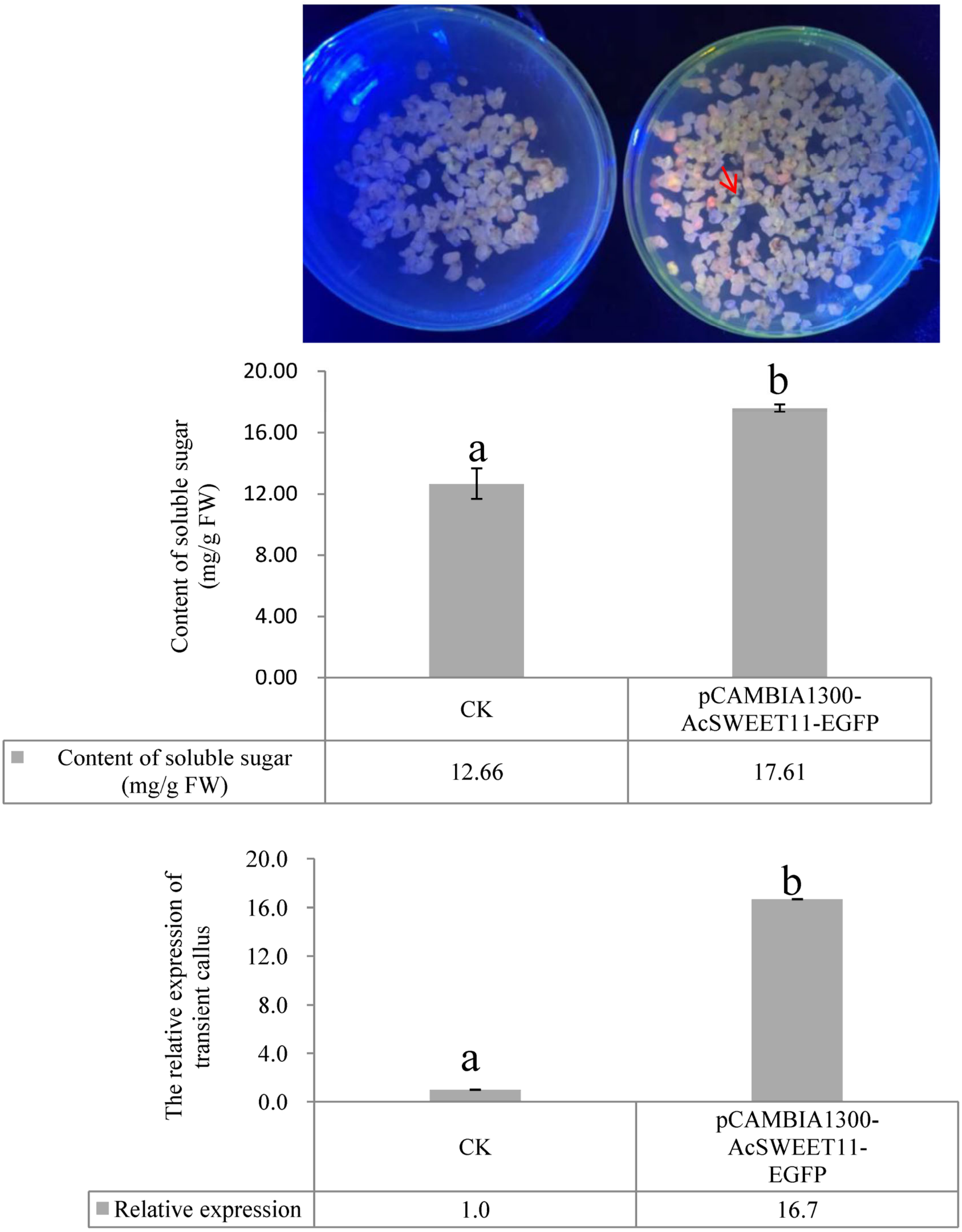

Figure S3. Soluble sugar content of pineapple embryogenic callus by agrobacterium-mediated vacuum infiltration. Arrow marked the callus of transient transfection with GFP. Different letters denoted significant difference with one-way ANOVA test by Tukey's test ($p < 0.05$).
